# Phosphorylation Hypothesis of Sleep

**DOI:** 10.3389/fpsyg.2020.575328

**Published:** 2020-10-02

**Authors:** Koji L. Ode, Hiroki R. Ueda

**Affiliations:** ^1^Department of Systems Pharmacology, Graduate School of Medicine, The University of Tokyo, Tokyo, Japan; ^2^Laboratory for Synthetic Biology, RIKEN Center for Biosystems Dynamics, Osaka, Japan

**Keywords:** NREM sleep, phosphorylation, kinase, proteomics, CaMKII

## Abstract

Sleep is a fundamental property conserved across species. The homeostatic induction of sleep indicates the presence of a mechanism that is progressively activated by the awake state and that induces sleep. Several lines of evidence support that such function, namely, sleep need, lies in the neuronal assemblies rather than specific brain regions and circuits. However, the molecular mechanism underlying the dynamics of sleep need is still unclear. This review aims to summarize recent studies mainly in rodents indicating that protein phosphorylation, especially at the synapses, could be the molecular entity associated with sleep need. Genetic studies in rodents have identified a set of kinases that promote sleep. The activity of sleep-promoting kinases appears to be elevated during the awake phase and in sleep deprivation. Furthermore, the proteomic analysis demonstrated that the phosphorylation status of synaptic protein is controlled by the sleep-wake cycle. Therefore, a plausible scenario may be that the awake-dependent activation of kinases modifies the phosphorylation status of synaptic proteins to promote sleep. We also discuss the possible importance of multisite phosphorylation on macromolecular protein complexes to achieve the slow dynamics and physiological functions of sleep in mammals.

## Introduction

Sleep is a fundamental property of the central nervous system. A stereotypical sleep-wake transition occurs from seconds to minutes in which nearly all cortical neural activity changes to the sleep-firing pattern. Some animal species to adopt different styles of the sleep-wake cycle called unihemispheric sleep observed in migratory birds (Rattenborg et al., 2016) and fur seals (Lyamin et al., 2017). Neural architectures for achieving sleep-wake transition also vary. Even jellyfish with a diffuse nervous system undergoes sleep-wake cycle (Nath et al., 2017), suggesting that sleep is not a specific function of animals with a central nervous system but a function that originated in an early stage of metazoan lineage.

Despite such anatomical differences in regulating sleep, the characteristics of sleep dynamics are well conserved throughout the phyla. Animals have homeostatic sleep control to ensure that they can keep a baseline sleep amount: if an animal spends more time in an awake state in one day, the reduced sleep amount is restored by taking an extra amount of sleep in the subsequent days (recovery sleep), suggesting that the amount of sleep loss is monitored and measured. The baseline daily duration of sleep varied depending on the animal species (Campbell and Tobler, 1984; Siegel, 2005) and even within inbred mice strains (Franken et al., 1999; Diessler et al., 2018): such differences are at least in part genetically inherited, suggesting that there are genetic factors that determine the normal amount of sleep per day.

In this review, we introduce recent progress in research, mainly focusing on the posttranslational regulation of proteins inside neurons, that reveal that phosphorylation plays an important role in the homeostatic regulation of sleep in mammals. Beyond the fact that phosphorylation is one of the most important modalities in the regulation of protein activity (Cohen, 2000, 2002; Nishi et al., 2014) and it is used to control the basic function of neurons such as long-term potentiation (LTP) (Soderling and Derkach, 2000), the global phosphorylation status in neurons appear to be controlled by sleep-wake cycles (Wang Z. et al., 2018; Bruning et al., 2019). Also, genetic evidence indicates that some classes of kinases have significant sleep-inducing effects (Funato et al., 2016; Tatsuki et al., 2016; Mikhail et al., 2017). Thus, phosphorylation may actively regulate the dynamics of sleep in mammals.

Sleep is composed of qualitatively different types of sleep modes, NREM and REM. The following sections mainly focus on NREM sleep because this mode of sleep constitutes most of the total sleep episodes (e.g., ∼90% in C57BL/6 mice), and several genetic studies discussed below reported the changes in the NREM sleep duration. To address how the phosphorylation-oriented view of sleep regulation complements the previous models of sleep control in mammals, we will begin with a brief overview of representative models proposed to explain the homeostatic regulation of sleep. In the following sections, we will discuss sleep in mammals, unless we explicitly mention the sleep of non-mammalian species.

## Hierarchical Organization of Circadian Clocks and Sleep Homeostasis

### Two-Process Model of Sleep-Wake Cycle

The two-process model, which has been the landmark for explaining the behavioral rhythmicity of the daily sleep-wake cycle, describes the relationship between sleep homeostasis, characterized by “Process S,” and the circadian clock, characterized by “Process C” (Borbely, 1982; Borbely et al., 2016). Process S represents the amount of sleep need, such that Process S increases in an awake state and decreases in a sleep state. When the level of Process S increases to the certain upper level (upper threshold), the animals go to the sleep phase, and when the level Process S decreases to the certain lower boundary (lower threshold), the animals turn to the awake phase. These upper and lower threshold levels are modeled to show a daily oscillation by the function of Process C. In the typical diurnal animals’ Process C, the peak of the lower threshold oscillation is around the morning and trough of it is around the night.

The mechanism driving Process C is a cellular circadian clock, in which the transcription-translation feedback loops (TTFLs) generate a cell-autonomous oscillation of transcription activity (Takahashi, 2017; Patke et al., 2020). The nearly 24-h rhythmicity of cellular activity is synchronized by a circadian clock in a specific brain region called the suprachiasmatic nucleus (SCN); if the SCN is disrupted, the synchrony of circadian clocks in each cell is lost, and the behavioral circadian rhythmicity no longer persists without the external stimulus (e.g., light-dark cycle), although the transcription rhythmicity in individual cells continues to some extent (Tahara et al., 2012). Therefore, the mechanism to maintain the 24-h time period lies in the molecular activity within the cell, and the synchrony among the cells is achieved by the hierarchical connection of intercellular interaction, including humoral factors (Silver et al., 1996).

The molecular mechanism of Process S is still unclear, but Process S is a good factor to evaluate and simulate the level of sleep need in human and rodents. The physiological metric well matched with the expected level of Process S is the slow-wave activity (SWA) of electroencephalogram (EEG) signal (typically from 0.5 to 4 Hz) (Borbely, 1982). The predicted Process S based on the two-process model well fits the daily dynamics of SWA and its response to sleep deprivation (Daan et al., 1984; Dijk et al., 1990a, b). The model successfully characterizes the different sleep phenotypes among inbred mice (Franken et al., 2001) and human individuals (Rusterholz et al., 2010). Furthermore, the model was also applied to classify the dynamics of the gene expression profile (Hor et al., 2019).

Although the original two-process model assumes that Process S monotonically decreases and increases in response to the sleep-wake state, the mechanism for the regulation of the sleep-wake cycle independent from the circadian clock component may include a non-linear feedback system that could work as an oscillator. A classic example is a phenomenon called internal desynchronization in humans, in which the sleep-wake rhythm has a different period length than the physiological rhythms driven by the circadian clock (e.g., rhythms in core body temperature) (Aschoff, 1965; Kronauer et al., 1982; Dijk and Czeisler, 1995). Under certain conditions, the sleep-wake cycle in rodents also exhibits autonomous behavioral rhythms that appear to be independent of the circadian clocks. An example is sleep-wake rhythmicity induced by the chronic application of methamphetamine (methamphetamine-sensitive circadian oscillator) (Honma et al., 1987). Methamphetamine-sensitive sleep-wake rhythmicity can be observed in the absence of both environmental time cues and canonical circadian clocks (e.g., SCN lesion or genetic background where canonical molecular factors essential for driving the circadian TTFLs were knocked out) (Honma et al., 1987, 2008; Masubuchi et al., 2001; Mohawk et al., 2009). A dopaminergic oscillator was proposed as the underlying mechanism of ultradian (∼6 h) behavioral rhythmicity observed in the absence of functional circadian clocks (Blum et al., 2014). Furthermore, the methamphetamine-sensitive rhythmicity is proposed as the period-modulated dopaminergic oscillator (Blum et al., 2014).

The molecular mechanism determining the basal sleep duration is also still unknown. In the two-process model, the duration of daily sleep is determined by the intercrossing point of Process S and Process C. Thus, the duration of baseline daily sleep should be altered by the manipulation of the increase/decrease rate of Process S or manipulation of the upper/lower threshold of Process C (Aeschbach et al., 1996). However, expected different response to the sleep deprivation by such manipulation was not observed between long sleepers and short sleepers in human (Aeschbach et al., 1996). Also, mice lines that have similar parameters for the increase and decrease of Process S show different daily sleep durations (Franken et al., 2001). These studies suggest that daily sleep duration is determined by a complex mechanism that is not covered by the simple two-process model.

In summary, although the two-process model is successful in explaining and predicting the organism-level sleep-wake behavior in mammals, the mechanisms underlying the behavior of Process S and the interaction between Process S and Process C are largely unknown. The understanding of molecular components involved in the homeostatic regulation of sleep will be a critical step to dissect, quantify and understand the feedback mechanism controlling the behavioral sleep-wake rhythmicity.

### Cortex Neuronal Activity During NREM Sleep

At the neuronal-level dynamics associated with the sleep-wake cycle, synchronous activity among neurons is a typical feature of NREM sleep, in which the cortical neurons exhibit a unique firing pattern that alternates between a bursting (up state) and resting (down state) phase at 0.5–1 Hz (slow oscillation). The slow oscillation occurs synchronously among the cortical neurons such that the synchronized bursting/resting pattern is thought to be the source of SWA observed in the EEG signal during NREM sleep. The synchronized bursting/resting pattern of cortical neurons during NREM sleep becomes less synchronized in response to the decrease of sleep need (Vyazovskiy et al., 2009).

The excitability of cortical neurons is modulated by the level of excitatory neurotransmitters (e.g., dopamine, norepinephrine, acetylcholine, etc.). The level of neurotransmitters is controlled through the inputs from several brain nuclei, including sleep-active (e.g., ventrolateral preoptic nucleus) and wake-active nuclei (e.g., locus coeruleus, tuberomammillary nucleus, Raphe) that are supposed to be mutually regulated (flip-flop model) (Saper and Fuller, 2017). The inputs can be external stimuli, such as wake-inducing caffeine, that are shown to affect the sleep-wake cycle through acting on the A2A receptor expressed at the nucleus accumbens (Lazarus et al., 2011).

The synchronous slow oscillation appears to be the inherent property of cortical neuron assemblies because the oscillation can be found in *ex vivo* cortical slabs/slices (Sanchez-Vives and McCormick, 2000; Timofeev et al., 2000). It was even shown that the cultured neurons exhibit a synchronous firing pattern similar to that of slow oscillation, and the synchronous firing pattern can be converted to asynchronous one by applying chemical(s) including excitatory neurotransmitters (Hinard et al., 2012; Kaufman et al., 2014; Saberi-Moghadam et al., 2018). Overall, the synchronous slow oscillation of cortical neurons can be modulated through multiple layers of neurotransmitters, brain circuits, and neuronal networks. However, the layer on which sleep need is encoded is still unclear.

Interestingly, several studies reported a phenomenon called local sleep, where a part of the cortex exhibits slow oscillation in a use-dependent manner even when the entire brain is in an awake state (Krueger et al., 2019). It is also known that the delta power of EEG (1–4 Hz) distributes heterogeneously and increases within the cortical areas that showed higher activity during the preceding awake period (Huber et al., 2006, 2007). These observations support the hypothesis of local use-dependent sleep, proposing that sleep need can be stored within a smaller number of neuron/glial cells and distributed throughout the cortex heterogeneously (Krueger and Obal, 1993; Krueger et al., 2008, 2019). The minimum cellular set for sleep homeostasis may be at the synapse level; the synaptic homeostasis hypothesis employs the sum of synaptic strength as the hallmark of the cellular component of sleepiness (Tononi and Cirelli, 2003, 2006) and is supported by the 3D electron microscopic quantification of synapses showing a decrease in the interface between axons and spines during sleep (de Vivo et al., 2017; Spano et al., 2019). In line with this view, cultured neuronal/glial cells develop a sleep-like firing pattern in a homeostatic manner upon the administration of wake-inducing stimulation (Saberi-Moghadam et al., 2018). If the sleep need does not rely on a specific set of brain circuits but on a smaller number of neuronal/glial networks, it is worth considering the possibility that molecular signaling inside the neurons, rather than the brain–circuit-level activity, is the direct source of the sleep need.

## Phosphorylation-Dependent Control of the Sleep-Wake Cycle

**FIGURE 1:**
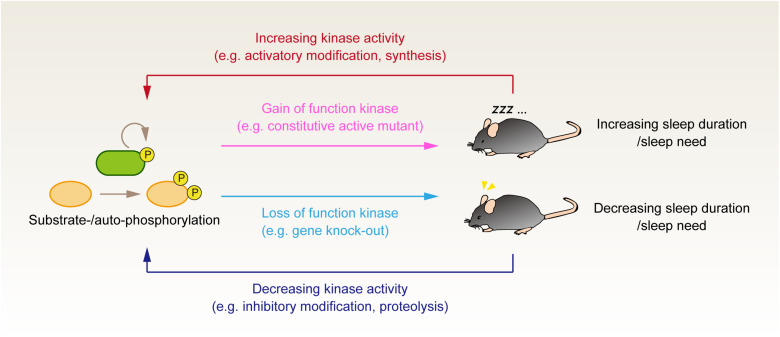
Genetic and phosphoproteomics approach to investigate the role of protein phosphorylation in the regulation of sleep. Genetic manipulation to either increase or decrease the kinase activity tests the causal effect of kinase activity on the animals’ sleep control. It would be valuable to evaluate whether the up- and downregulation of the same kinase activity affects the animals’ sleep phenotype bidirectionally. Although such kinase activity is not fully established, this criterion would discriminate whether the kinase activity serves as an external input to the core homeostatic process or if the kinase is involved in the core component of sleep homeostasis. Phosphoproteomics analysis provides complementary information to evaluate whether the kinases’ activity correlates with the expected sleep pressure in vivo. Note that this figure depicts the case of sleep-promoting kinases; when the kinases serve as a wake-promoting factor, the relationship between enzyme activity and sleep phenotype would be the opposite direction (i.e., loss of wake-promoting kinase would result in the increased sleep duration).

To investigate candidate genes involved in the molecular signaling pathway responsible for sleep homeostasis control in mammals, researchers have been conducting various genetic studies to find genetic manipulations that alter the animals’ baseline sleep duration, SWA and its response to sleep deprivation (Jan et al., 2020). To validate whether the candidate gene product controls the animals’ sleep in physiological condition, it is also critical to confirm that the activity of candidate protein is changed in accordance with the sleep-wake cycle or sleep-deprived situation. In the following sections, we introduce recent genetic and biochemical studies indicating the importance of protein kinases in inducing sleep (Figure 1). Because the activity of kinases is often regulated by phosphorylation modifications, changes in the phosphorylation state of the kinases have also been investigated quantified along with the sleep-wake cycle.

### CaMKIIα/CaMKIIβ

*Camk2a/Camk2b* were found as sleep-promoting kinases by a reverse-genetic screening of sleep-regulating genes using the CRISPR/Cas9 system in mice (Tatsuki et al., 2016). Embryonic knockout of *Camk2a* and *Camk2b*, but not *Camk2d* and *Camk2g*, leads to a significant decrease in sleep duration per day. *Camk2a/Camk2b* encodes calcium-/calmodulin-dependent kinase II (CaMKII) α/β, expressed widely in the brain. Because the knockout mice had shorter sleep duration, CaMKIIα/CaMKIIβ appears to have a role in promoting sleep. It should be noted that several studies suggest the wake-/REM-associated roles of CaMKII in specific brain regions (Stack et al., 2010; Datta et al., 2011; Cui et al., 2016), and embryonic knockout study alone cannot identify the brain area and cell type responsible for the phenotype. Even for the *Camk2a/Camk2b*, which are well understood as the enriched expression in (excitatory) neuron cells, both CaMKII isoforms are also expressed in astrocytes (Takeuchi et al., 2000). In addition, embryonic knockout of *Camk2a* and *Camk2b* would be accompanied by the effect on neuronal development (Wayman et al., 2008) that may have a chronic effect on the sleep-wake cycle. Therefore, the exact mechanism of sleep induction by CaMKIIα/CaMKIIβ needs to be clarified through the post-natal brain-area-/cell-type-specific perturbation of these kinases.

CaMKII has a unique self-regulatory mechanism depending on the auto-phosphorylation activity. CaMKII is typically activated by the binding of calcium/calmodulin after synaptic stimulation. The active CaMKII then auto-phosphorylates the CaMKII itself. Autophosphorylation occurs on T286 (CaMKIIα) or T287 (CaMKIIβ). The T286/287 phosphorylation keeps the kinase in an active form, even in the absence of Ca^2+^/calmodulin (Miller et al., 1988). Several studies in mice demonstrated that the phosphorylation of T286/287 is increased after sleep deprivation or during the awake/dark phase (Vyazovskiy et al., 2008; Diering et al., 2017; Wang Z. et al., 2018; Bruning et al., 2019), suggesting that the kinase activity of CaMKIIα/CaMKIIβ is upregulated in these conditions where sleep need would be elevated.

CaMKII is one of the best-studied kinases for its critical role in regulating synaptic plasticity (Coultrap and Bayer, 2012; Bayer and Schulman, 2019), and its regulatory mechanism has been extensively studied. The autophosphorylation at T286/287 is required for LTP (Giese et al., 1998), although the Ca^2+^/calmodulin-independent kinase activity is not needed for maintaining long-term memory (Buard et al., 2010). After the activation CaMKII, autophosphorylation continues to the other multiple residues. Autophosphorylation at T305/T306 (CaMKIIα) and T306/T307 (CaMKIIβ) are well known downstream autophosphorylation targets. The subsequent T305/T306 phosphorylation inhibits the binding of Ca^2+^/calmodulin to the kinase (Colbran and Soderling, 1990), and disruption of the auto-inhibitory mechanism also affects whether the activated CaMKII facilitates or depresses the synaptic strength (Pi et al., 2010b). The roles of these multisite autophosphorylation events in regulating sleep duration remain a mystery. Given the multi-step regulation encoded as a protein function of CaMKII, it may be worth investigating how CaMKIIα/CaMKIIβ with different phosphorylation states control the sleep phenotype differently.

### SIK3

The second discovery of sleep-promoting kinase in mammals was derived from large-scale forward-genetic screening (Funato et al., 2016). The screening of >8,000 mice generated by ENU-based mutagenesis identified the *Sleepy* mutant showing prolonged NREM sleep. The extended NREM sleep is caused by a mutation that leads to the exon skipping the salt-inducible kinase (SIK) 3. The exon skipping is suggested to be the gain-of-function of SIK3; thus, SIK3 is also a member of sleep-promoting kinases.

SIK3 activity is regulated by phosphorylation by other kinases (Sakamoto et al., 2018). The exon skipped in the *Sleepy* mutant involves a phosphorylation site, S551, modified by protein kinase A (PKA). SIK3 also has several phosphorylation sites, some of which have higher phosphorylation levels because of sleep deprivation or the *Sleepy* mutation (Funato et al., 2016). Among the multiple phosphorylation sites, S551 appears to be critical for promoting NREM sleep, because the CRISPR-mediated embryonic introduction of S551A point mutation reproduced the effect of sleep promotion (Honda et al., 2018). By using the similar CRISPR-mediated targeted mutagenesis, it was demonstrated that the PKA-targeted site plays a role in the NREM sleep promotion in other SIK family proteins, SIK1 and SIK2 (Park et al., 2020). Interestingly, the role of SIK family in the sleep regulation appears to be conserved in invertebrate animals because the temporal induction of S563A (corresponding to the S551A in mice) SIK3 mutant also increase sleep duration in *Drosophila* (Funato et al., 2016).

However, the exact role of phosphorylation at this residue is still unclear. This is because not only S551D, mimicking the phosphorylated status of Ser residue, but also S551A, mimicking the unphosphorylated state of the residue, resulted in a similar phenotype of *Sleepy* exon skipping (Honda et al., 2018). Although D/A mutations do not always mimic the phosphorylated/unphosphorylated status of Ser residue, the result implies that the reversible phosphorylation that occurs on this site may not account for the reversible regulation of sleep duration. The role of SIK3 and the phosphorylation-dependent regulation of SIK3 in regulating natural sleep will need to be assessed through the sleep phenotype of SIK3 knockout mammals, which has not been reported to date. Notably, *Sik3*-null mutant of *C. elegans* and hypomorphic *Sik3* mutant of *Drosophila* have decreased amount of sleep-like quiescence behavior, consistent with the sleep-promoting role of SIK3 in mammals (Funato et al., 2016).

### ERK1/ERK2

Extracellular signal-regulated kinase (ERK) is also a sleep-promoting kinase. This was first suggested in *Drosophila*, where pan-neuronal expression of the kinase-active form of ERK increases sleep duration and application of ERK inhibitor reduces sleep (Vanderheyden et al., 2013). However, the hypomorphic ERK mutant flies show sleep duration similar to wild type flies (Vanderheyden et al., 2013), raising the question of how much of the basal sleep duration is under ERK-specific control. The role of mammalian ERK as a sleep-promoting kinase was then clearly demonstrated in mice (Mikhail et al., 2017). Embryonic knockout of ERK1 and conditional ERK2 knockout largely in cortical neurons both result in a decrease in NREM sleep duration. The administration of ERK1-/ERK2-selective inhibitor (U0126) also decreases NREM sleep, suggesting that the ERK1/ERK2 regulate sleep independent of neurodevelopmental effect. Consistent with this observation, SWA was decreased in the U0126-treated mice. Conversely, the level of phosphorylated ERK1/ERK2 (kinase-active ERK1/ERK2) is higher in the arousal state than that in the NREM sleep.

The role of mammalian ERK as a sleep-promoting kinase may lie in the control of gene expression associated with sleep-wake cycle. This notion was demonstrated through unique studies using cultured cortical neurons, which show electrophysiological activity similar to slow oscillation, together with the homeostatic recovery response to the administration of chemical/electrical stimulation (Hinard et al., 2012; Saberi-Moghadam et al., 2018). The cortical neurons also show altered gene expression by chemical stimulation mimicking the awake state. Notably, inhibition of ERK signaling antagonized the changes in gene expression caused by the wake-mimicking stimulation (Mikhail et al., 2017).

### Sleep Need, SWA, and Recovery Sleep

Does perturbation of the sleep-promoting kinases affect the indicators of Process S? Significant reduction of daily sleep amount observed in *Camk2a*/*Camk2b* knockout mice suggests that CaMKIIα/CaMKIIβ is involved in the mechanism determining the basal sleep duration (Tatsuki et al., 2016). In the study of Tatsuki et al., the sleep phenotype was recorded using a respiratory-based system (Sunagawa et al., 2016), which can non-invasively measure sleep and awake with high accuracy, but, to date, cannot discriminate NREM and REM sleeps. Accordingly, the SWA and its response to sleep deprivation were not validated in *Camk2a/Camk2b* knockout mice, and they ought to be investigated in a future study. As for SIK3, *Sleepy* mice have an increased level of SWA, suggesting that the mice maintain a higher level of sleep need, resulting in the longer sleep phenotype (Funato et al., 2016). *Sleepy* mice were also shown to be susceptible to sleep deprivation. Taken together, the *Sleepy* mice may have accelerated accumulation and/or decelerated clearance of Process S. On the other hand, knocking out ERK1 or ERK2 reduces NREM sleep duration to the same extent in both normal and recovery sleep (Mikhail et al., 2017). Interestingly, inhibition of ERK1/ERK2 activity does not result in the changes in the delta power of EEG. Thus, in contrast to the case of SIK3, ERK1/ERK2 may not be involved in the accumulation process of sleep need but rather in the induction of NREM sleep downstream of the sleep need.

The examples of SIK3 activation and ERK1/ERK2 inhibition imply that these kinases are involved in the regulation of normal sleep duration but do not significantly impair the induction of recovery sleep. This is in stark contrast to the phenotype caused by the inhibition of gliotransmission. Inhibition of gliotransmission by the expression of the dominant-negative form of SNARE leads to the suppression of rebound sleep after sleep deprivation and the suppression of acute sleep induced by the inflammatory response (Halassa et al., 2009; Nadjar et al., 2013). Under this condition, however, the basal level of sleep duration does not change. At this point, we shall revisit the essence of the two-process model: the two-process model summarizes the dynamics of sleep need and recovery sleep as a single scaler “Process S,” but in the molecular view, a mechanism that maintains a homeostatic daily amount of sleep and other mechanisms that induce additional sleep pressure upon an unusual situation, such as significant sleep deprivation or inflammatory response, may be separated. Some of the chemicals secreted outside the cell called sleep-inducing substances (SISs) appear to be involved in the latter mechanism. For example, knockout of the adenosine A1 receptor impairs the increase of SWA after sleep deprivation without affecting total sleep (Bjorness et al., 2009). The knockout of adenosine A2 also does not show a significant effect on normal sleep but diminishes the wake-promoting effect of caffeine (Huang et al., 2005). The knockout of receptors for SISs related to the inflammatory responses (e.g., TNF-α) also results in normal sleep duration, although the SWA during NREM sleep is paradoxically increased in TNF-α receptor knockout mice (Fang et al., 1997; Kaushal et al., 2012). Compared with these SISs, the sleep-promoting kinases appear to have a role in regulating normal sleep duration.

The sleep-promoting kinases may be involved in the interplay between the circadian clocks and sleep control. Although circadian clocks and sleep homeostasis can run independently, circadian genes affect the sleep-wake behavior and vice versa (Franken and Dijk, 2009). In the case of sleep-promoting kinases, all of the abovementioned kinases are suggested to have a role in regulating circadian clocks. CaMKII plays a critical role in the robust oscillation of the circadian clock in the SCN slice through the phosphorylation of a circadian transcription factor called CLOCK (Kon et al., 2014). Notably, inhibition of CaMKII also attenuated the circadian oscillation of non-neuronal cultured cells, indicating that CaMKII plays a role independent of neural firing for circadian rhythmicity (Kon et al., 2015). SIK3 is shown to be involved in regulating circadian period. The knockout mice for *Sik3* show several abnormalities in circadian rhythmicity, including a longer circadian period in the behavioral rhythmicity and impaired synchrony of individual cells in the SCN (Hayasaka et al., 2017). A molecular link between SIK3 and circadian clocks could be the SIK3-dependent phosphorylation and degradation of PER2 (Hayasaka et al., 2017). Abnormal circadian rhythmicity and PER protein dynamics are also observed in *Drosophila* lacking *Sik3* function (Fujii et al., 2017). ERK was reported to be involved in the resetting process of circadian clocks (Akashi and Nishida, 2000). In addition, chemical screening studies have identified ERK inhibitors as potent perturbator of cellular circadian oscillation (Isojima et al., 2009; Kon et al., 2015), although part of ERK inhibitors also targets casein kinase 1 (CKI) *δ*/*ε*, which is a critical kinase for the circadian period determination (Isojima et al., 2009). There are several pathways connecting ERK activity and the circadian molecular feedback loop, mainly through the transcription control by ERK of the downstream signaling cascade (Goldsmith and Bell-Pedersen, 2013). One example is the transcription factor *Dpb*, which composes the circadian TTFLs. The *Dpb* expression is suppressed in sleep-deprived rats (Cirelli et al., 2004) as well as cultured cortical neurons stimulated by wake-mimicking chemicals (Mikhail et al., 2017).

In summary, sleep-promoting kinases markedly affect normal sleep duration. The kinases may shape the molecular dissection of the mechanism involved in Process S and mechanism determining basal sleep duration, and reveal the difference between sleep need to maintain daily sleep duration and the induction of recovery/inflammatory sleep. The kinases may also work as a hub to connect sleep homeostasis and circadian regulation.

## Sleep-Wake-Dependent Control of Phosphoproteome Profile

### Uncoupling Between Transcriptome and Proteome Profiles

**FIGURE 2:**
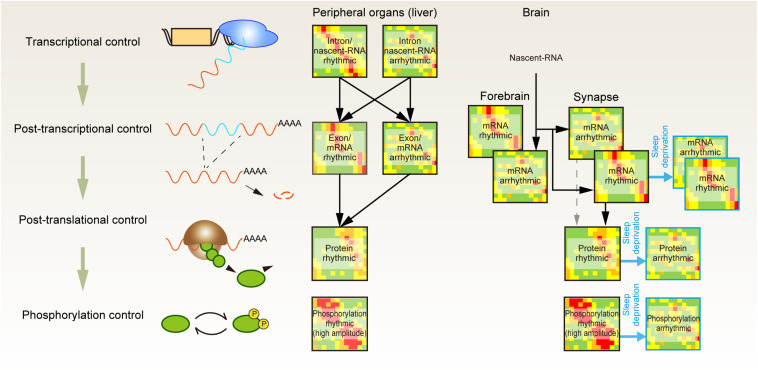
Posttranscriptional, posttranslational, and protein phosphorylation shape the daily rhythmicity of protein action. Daily rhythmicity of mRNA and protein abundance is created not only by the timing of transcription initiation but also through multiple layers of posttranscriptional and posttranslational regulation. The continuous elevation of sleep pressure predominantly attenuates the rhythmicity observed in protein and phospho-protein abundance at the synapse.

A complementary approach for investigating the importance of protein phosphorylation is to quantify protein abundances and their phosphorylation status along with the sleep-wake cycle (Figure 1). It is well understood that most of the transcriptome profile is under the control of circadian clocks (Reppert and Weaver, 2002). Accordingly, researchers have attempted to find genes in which the expression pattern shows daily rhythmicity and changes upon the accumulation of sleep need (e.g., sleep deprivation condition) (Elliott et al., 2014; O’Callaghan et al., 2019). However, advances in next-generation sequencing and mass spectrometer-based -omics technologies indicate that the gene expression pattern does not always reflect the expression pattern of gene products (Figure 2). Deep sequencing allows researchers to compare intron and exon cycling genes in the liver and has revealed that many of them are not overlapped, indicating that the rhythmicity of a significant amount of mRNA expression is controlled through posttranscriptional regulation (Koike et al., 2012). A similar conclusion has been obtained by comparing nascent-RNA sequencing (Menet et al., 2012), RNA polymerase II occupancy (Le Martelot et al., 2012), and modeling studies aiming to clarify the contribution of mRNA degradation to the rhythmicity of mRNA abundances (Wang J. et al., 2018).

A quantitative proteomics study of liver samples further revealed the uncoupling between the peak phase of cycling proteins and their corresponding mRNAs. Moreover, ∼20% of rhythmic protein may not be accompanied by rhythmic mRNA expression, indicating the importance of posttranscriptional mechanisms to generate the rhythmic protein expression (Robles et al., 2014). At the posttranslational level, rhythmic protein activities appear to be enhanced by protein phosphorylation. Comparison of the proteomic and phosphoproteomic profiles showed that the amplitude of the rhythmic component (protein abundance or phosphopeptide abundance) was several times higher on average for the phosphorylated peptide (Robles et al., 2017). Therefore, the oscillatory dynamics of protein function seem to be generated through not only promoter-driven gene expression but also posttranscriptional and posttranslational control and enhanced at the layer of protein phosphorylation.

### Impact of Sleep Need on Proteomic and Phosphoproteomic Profile

Modern phosphoproteomic analysis has also been applied to determine factors responding to sleep deprivation. An analysis of postsynaptic density (PSD) fraction identified several proteins and phosphopeptides showing different abundances between light and dark phases (Diering et al., 2017). Among them, the level of CaMKIIα with activated autophosphorylation (T286) was found to be approximately twofold higher in the dark phase at which the nocturnal mice spend much of its time in an awake state. Thus, this kinase seems to have higher activity during the awake phase. Interestingly, one of the other phosphorylation residues of CaMKIIα (T310) is lower in the dark phase, suggesting that not only the amount of phosphorylation but also the pattern of multisite phosphorylation varies, depending on the sleep/awake state.

The rhythmic dynamics of each layer of -omics profiles can be generated directly by circadian TTFLs or indirectly as a consequence of clock-controlled changes in cellular activity, such as sleep-wake transitions. As for the case of proteomics and phosphoproteomics profiles in the brain, a recent study shows that the latter profile is dominant. Time-course comparison between synaptic transcriptome and proteome profiles found a striking effect of sleep deprivation on the daily rhythmicity of synaptic proteins (Noya et al., 2019). This study revealed that 67% of synaptic transcripts are rhythmically expressed in the synaptosome, but in the entire forebrain fraction, most of the transcripts are not rhythmic. Therefore, the source of rhythmicity for the synaptic transcripts lies in the posttranscriptional control at synapses. In addition, 27% of such rhythmic transcripts still remain rhythmic even when the mice are challenged to 4-h sleep deprivation prior to each sampling time point. However, the sleep deprivation protocol diminishes the rhythmicity in the protein abundances, which are otherwise observed in accordance with the rhythmic transcripts. Therefore, translational and/or posttranslational control at the synapse is dominated by sleep-wake states, and 4-h sleep deprivation locks the proteomic profile into a sleep-deprived (or awake) state. Assuming that most of the cellular functions are determined by the gene products rather than the expressed RNA itself, the effect of sleep deprivation on protein abundance that is strong enough to mask the rhythmic transcription indicates that the temporal synaptic status is largely determined by the sleep-wake state rather than the circadian phase.

A phosphoproteomic profile of the synaptosome further revealed circadian-phase-specific activation of kinase signaling (Bruning et al., 2019). In the synaptosome, ∼30% of quantified phosphopeptides show daily rhythmicity. Most of the phosphorylation rhythm is supposed to be generated by the action of kinases (or phosphatases). This is because the majority of rhythmic phosphopeptides are derived from proteins that do not exhibit daily oscillations. Even for minor rhythmic phosphopeptides derived from rhythmic proteins, the amplitude of oscillations is much higher in phosphopeptides. The computational prediction assigned CaMKII and PKC as kinases that are activated at the transition phase from sleep to awake, suggesting that the activities of these kinases are regulated rhythmically. The Western blot against these kinases with phosphorylation that marks the kinase-active forms showed an interesting daily oscillation pattern: the active CaMKIIα (phosphorylated T286; autophosphorylation site) continuously increases during the awake period and then decreases during the sleep period. This behavior mirrors the anticipated dynamics of Process S and is well matched with the expected role of CaMKII as a sleep-promoting kinase. The active PKC (phosphorylated S660; autophosphorylation site) behaves symmetrically to CaMKII: the (auto)phosphorylated PKC increases during the sleep phase and decreases during the awake phase. This reciprocal activation of PKC and CaMKII is of particular interest in that both kinases are involved in the induction of LTP (Malenka et al., 1989; Malinow et al., 1989); the phosphorylation dynamics suggests that this PKC has a mutually exclusive role with CaMKII in sleep-wake regulation, although the conclusive role of PKC in the regulation of the sleep-wake cycle needs to be clarified.

Similar to the case of rhythmicity in protein abundances, the rhythmic phosphopeptide dynamics are dominated by sleep-wake transition rather than the circadian clocks (Bruning et al., 2019). The 4-h sleep deprivation applied prior to each sampling point across the day eliminated the rhythmicity of ∼98% otherwise rhythmic phosphopeptides. In agreement with this, the peak points of rhythmic phosphopeptides were accumulated at the transition of either the sleep-to-wake or wake-to-sleep phase. The drastic changes in the phosphoproteomic profile imply that the phosphorylation status can be a molecular representation of sleep need. However, sleep deprivation increases not only the level of sleep need but also the preceding awake duration. Because some phosphorylation responds rapidly to the prior sleep/wake state (Mikhail et al., 2017), distinguishing whether the changes in the phosphorylation status are caused by the increased sleep need or signals associated with the increased awake state (e.g., visual input) is difficult. Recently, *Sleepy* mice were used for phosphoproteomic analysis to study this problem (Wang Z. et al., 2018). This study compared the proteomic and phosphoproteomic profiles caused by sleep deprivation and the *Sleepy* mutation. Both perturbations are supposed to increase the sleep need, but the behavioral effects caused by these perturbations are opposite: *Sleepy* mice should spend a longer time in the sleep state, while sleep deprivation disturbs the sleep state. The results demonstrated that both perturbations similarly increase phosphorylated proteins without much increase in the amount of total (non-phosphorylated) protein; indeed, the researchers have a series of phosphorylated proteins, named as the sleep-need-index-phosphoproteins (SNIPPs), that increase in both sleep-deprived and the *Sleepy* mice. Many SNIPPs are involved in the synaptic regulation and, interestingly, include CaMKII. CaMKII activation during sleep deprivation may simply be due to increased synaptic input during the wake period, but similar activation of CaMKII in the *Sleepy* mice with short awake duration strongly suggests that CaMKII activity is associated with an increased need for sleep.

There could be several (not mutually exclusive) reasons for the global changes in the phosphorylation states. The first possibility is that altered neuronal excitability and synaptic inputs associated with different arousal state affect several types of kinases as predicted in phosphoproteomic studies (Wang Z. et al., 2018; Bruning et al., 2019). Because current bioinformatics analyses can only predict kinase-substrate relationships for a limited number of substrates (Needham et al., 2019), perturbations to multiple kinases may result in changes in the phosphorylation state of a larger number of residues than one would expect based on current biochemical information.

The second possibility would be that the sleep-wake cycle may affect protein phosphatase activity. Protein phosphorylation levels in cells are reversibly controlled by the balance between kinase and phosphatase activities. Phosphatases had been generally considered as less-specific promiscuous enzymes because they act on many substrate sequences. This view, however, is being revised as phosphatase-substrate specificity is controlled through various combinations of catalytic and regulatory subunits (Moorhead et al., 2007; Sacco et al., 2012). Several regulatory subunits appear to be involved in the mammalian sleep-wake control. The phenotype catalog of gene knockout mice developed by the International Mouse Phenotyping Consortium (Brown and Moore, 2012a, b; Ring et al., 2015) reported that knockout of *Ppp1r9b* (protein phosphatase 1 regulatory subunit 9B) results in the reduced sleep duration per day analyzed by the piezoelectric-sensor-based activity recording^1^ (Jan et al., 2020). In the transcriptome analysis of rodents, several catalytic and regulatory subunits of protein phosphatases were identified as genes whose expression levels are affected by sleep deprivation (Cirelli et al., 2004; Mackiewicz et al., 2007; Maret et al., 2007). Interestingly, knockout of calcineurin, a Ca^2+^/calmodulin-dependent protein phosphatase, and its regulator *sarah* in *Drosophila* resulted in a significant reduction of sleep duration (Nakai et al., 2011; Tomita et al., 2011), suggesting a possible connection between Ca^2+^ signaling and protein phosphorylation status in the sleep control mechanism. The expression of constitutive active calcineurin leads to controversial results in two studies (Nakai et al., 2011; Tomita et al., 2011), potentially indicating the importance of the proper level of phosphatase activity.

The third possibility is about protein turnover control. Because newly synthesized proteins are unphosphorylated form, increased protein turnover rate (e.g., increased proteolysis activity and increased transcription/translation activity) would help to decrease the phosphorylation level. It has been shown that the genes involved in the “macromolecular biosynthesis” (including protein synthesis and transport) are upregulated in the sleep phase (Mackiewicz et al., 2007). If such biosynthesis machinery contributes to *refresh* proteins, it can be assumed that the rate of protein turnover alters the global phosphorylation status in sleep-wake cycle. Stable-isotope labeling technique can be used to evaluate the omics-scale protein turnover rate in neurons (Dorrbaum et al., 2018), and it would be worth investigating the proteome-wide changes in the protein turnover rate along with the sleep-wake cycle. In addition, the turnover rate of specific proteins is often controlled through the ubiquitin-proteasome pathway. There are some studies suggesting the involvement of the proteasome system in the sleep-wake control. Mouse with a knockout of *Ube3a*, an E3 ubiquitin ligase, at maternal allele is a model animal for Angelman syndrome and cause a reduced SWA in NREM sleep and in response to sleep deprivation (Colas et al., 2005; Ehlen et al., 2015). In *Drosophila*, impaired activities of *Cul3* and *insomniac*, an E3 ubiquitin ligase and its adaptor, respectively, resulted in the reduced sleep amount (Stavropoulos and Young, 2011; Pfeiffenberger and Allada, 2012).

Overall, phosphoproteomics studies demonstrate that sleep-wake transition dominates the phosphorylation status of synaptic proteins. The clever strategy used to find the SNIPPs strengthens the hypothesis that the phosphorylation status of protein marks the level of sleep need. The proteomics measurements alone reveal only the correlation between phosphorylation and the expected level of sleepiness; however, given the fact that phosphoproteomics analysis also suggests that CaMKII activity increases with the transition from awake to asleep and with the stimulation of sleepiness by two independent perturbations, sleep-promoting kinases, which have been shown to have a causal effect on sleep duration, can be reasonably regarded as molecules that promote sleep according to the accumulation of the awake period *in vivo*.

## Phosphorylation Hypothesis of Molecular Sleepiness: A Perspective

### Connecting Physiological Context to the Sleep Regulation

**FIGURE 3:**
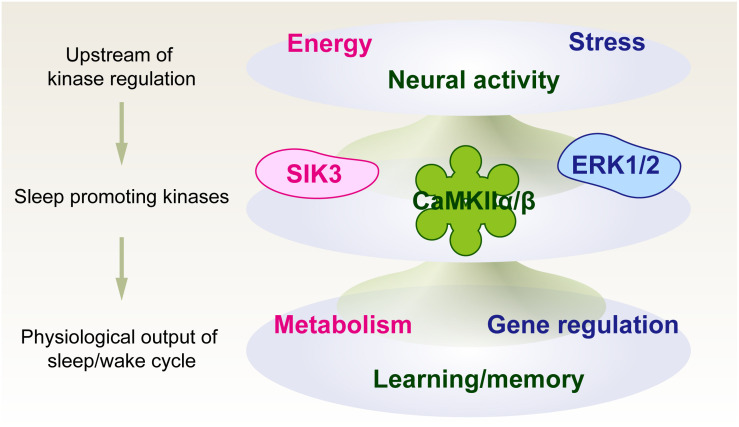
Phosphorylation hypothesis of molecular sleepiness. Sleep-promoting kinases may play important roles in integrating several signals that affect sleep-wake dynamics. The activities of sleep-promoting kinases, in turn, may affect sleep-wake dynamics as well as physiological functions associated with neural activity.

We first propose a kinase-centric view of sleep need, namely, a phosphorylation hypothesis of molecular sleepiness, based on the finding of the sleep-promoting kinase CaMKIIα/CaMKIIβ (Tatsuki et al., 2016). This hypothesis has been strengthened by the subsequent finding of other sleep-promoting kinases as well as phosphoproteomic investigations of molecular dynamics according to the sleep-wake cycle and its disturbance. The identified sleep-promoting kinases are involved in a number of cellular functions rather than just a specific sleep-promoting event; the example includes involvement of CaMKIIα/CaMKIIβ in the regulation of learning and memory (Coultrap and Bayer, 2012), ERK for stress responses (Coulthard et al., 2009), and SIK for the regulation of metabolic processes (Sakamoto et al., 2018). Because of these multiple functions of the sleep-promoting kinases, the kinase-centric view of sleep need opens an avenue to reevaluate the physiological and biochemical aspects of sleep control by integrating multiple inputs and outputs associated with sleep-wake control (Figure 3).

**FIGURE 4:**
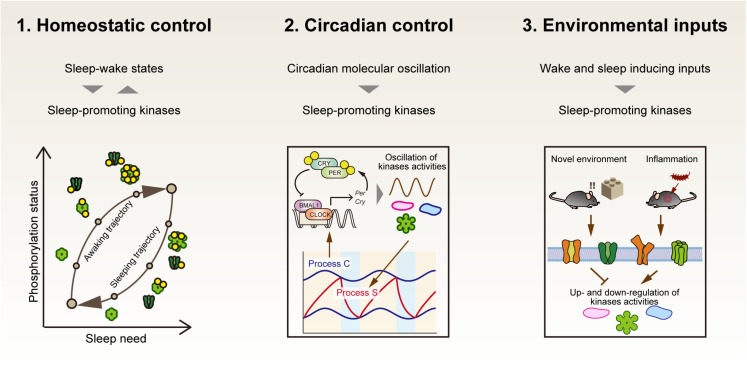
Three properties that the central factor responsible for sleep homeostasis should have. If sleep-promoting kinases compose a core mechanism of sleep homeostasis, first, the dynamics of kinase activity would correspond to the level of sleep need. Second, the kinase activity would be directly or indirectly regulated by circadian clocks as an interface of sleep homeostasis and circadian clocks. Third, the kinase activities may mediate various external signals to affect the arousal state of animals.

If protein activities modified by phosphorylation are the *de novo* molecular signature of sleep need, at least the following aspects should be explained by critical phosphorylation events (Figure 4). First, the phosphorylation status should be directly related to the homeostatic regulation of sleep. This will be achieved by demonstrating a causal sleep-promoting effect of a specific phosphorylation event(s) that is, in turn, regulated by the sleep-wake cycle (feedback regulation). Second, given the strong effect of circadian clocks on the behavioral sleep-wake cycle, the specific phosphorylation events should be linked with circadian clock control. Such phosphorylation events will account for the unidentified molecular interface between sleep homeostasis and circadian clocks. In addition, given the fact that the sleep-wake cycle is strongly affected by external stimuli, the third aspect will be that the specific phosphorylation events should also respond to the environmental and internal signals that promote either sleep or wakefulness.

The most evident signal to regulate sleep is the neuronal input, which can be directly transduced and integrated into CaMKII activation by the intracellular Ca^2+^ signaling. This would be straightforward: activation of sleep-promoting CaMKII is mediated by the increased neuronal spikes presumably associated with the awake state. Factors that may transduce the wake-associated information to the kinase are not limited to the synaptic input. Several monoamines working as excitatory neuromodulators (e.g., norepinephrine or dopamine) that promote wakefulness (Fuller et al., 2011) may also alter the activity of CaMKII by modulating the excitability of neurons. The SIK family is controlled by the multisite phosphorylation caused by elevated cAMP and subsequent activation of PKA. The neuronal cAMP level is modulated by several G protein-coupled receptors, including receptors of the excitatory monoamine neuromodulators. Although PKA has been known to mediate an awake-promoting effect caused by octopamine neurotransmitter in *Drosophila* (Hendricks et al., 2001; Crocker et al., 2010), the PKA-SIK pathway in mammals might be involved in the sleep-promoting effect of neurotransmitters which activates the cAMP in cortical neurons. On the other hand, ERK is activated by a wide range of cell signaling from cell differentiation to cell death (Coulthard et al., 2009). A potential pathway related to sleep control is stress-induced activation of ERK, which may be triggered by sleep deprivation that is known to be associated with the elevation of inflammatory responses (Clinton et al., 2011). If a part of these signals activates the sleep-promoting kinases, the kinases may bridge the cellular signaling to the induction of sleep.

Alternatively, because sleep-wake transition and sleep deprivation alter the phosphorylation status of sleep-promoting kinases, the altered kinase activities would affect not only the sleep regulation, but also cellular functions associated with each sleep-promoting kinase. In other words, sleep-promoting kinases may serve as hubs for cellular signaling cascades linking environmental/internal signals that regulate sleep and cellular functions that are regulated by sleep. For example, the well-understood function of CaMKII is its role in regulating synaptic plasticity (Coultrap and Bayer, 2012; Bayer and Schulman, 2019), and in the hippocampus, the altered phosphorylation status of CaMKII affects the synapse size (Pi et al., 2010a). The sleep-wake cycle would also affect synaptic strength. However, whether NREM sleep contributes in the direction of strengthening or weakening synaptic strength, or both, is still a matter of debate (Puentes-Mestril and Aton, 2017; Klinzing et al., 2019). Several studies showed that synaptic strength is strengthened (Chauvette et al., 2012) and spine formation at a specific set of dendrite is promoted (Yang et al., 2014) during NREM sleep. These synaptic enhancements during NREM sleep would depend on the synaptic inputs during the awake phase that precedes the sleep episode. By contrast, other lines of evidence indicate that the size of the axon-spine interface in several cortical areas and hippocampal CA1 region are, on average, decreased in the sleep phase compared with the awake phase (de Vivo et al., 2017; Spano et al., 2019). It is also demonstrated that REM sleep but not NREM sleep is important for the selective elimination of newly formed synapses depending on the dendritic Ca^2+^ spikes (Li et al., 2017). To uncover cellular and sub-cellular responses upon the sleep induction, local and molecular induction of sleep pressure would be a critical experiment. Thus, the sleep-promoting kinases would be key molecules and pertabator to uncover the molecular mechanism of reciprocal interaction between synaptic function and sleep-wake regulation.

### Connecting Different Timescales

The other perspective concerns the regulation of different timescales. The phosphorylation reaction typically occurs in a timescale of seconds to minutes, while the accumulation of sleep need should occur on a scale of minutes to hours (or even days) (Franken et al., 2001; Van Dongen et al., 2003; Wisor and Clegern, 2011). Is it possible that simple phosphorylation inside a neuron can count and integrate events that occur on such a slow timescale?

Researchers have been tackling this problem for CaMKII in terms of the regulation of synaptic plasticity and memory function. Although the activity of CaMKII is elevated continuously by repeptide synaptic inputs, the integrated CaMKII activity seems not to be maintained on more than a minutes scale (Chang et al., 2017). Nonetheless, it has been well understood that CaMKII kinase activity and proper autophosphorylation at T286 and other sites are important for types of neuronal plasticity and learning, including hippocampal LTP and spatial learning (Mayford et al., 1996; Giese et al., 1998; Elgersma et al., 2002; Yamagata et al., 2009). The question is how the relatively short term event, such as the autophosphorylation at CaMKII T286, which occurs within few minutes after the simulation (Coultrap and Bayer, 2012), can be converted to cellular outputs with slower dynamics. Apparently, changes in the synaptic strength and memory consolidation involve cellular events downstream of CaMKII kinase action, including the translocation of CaMKII and subsequent modification of synaptic architecture (Lisman et al., 2012). CaMKII itself can also encode information spanning a longer time duration because this kinase undergoes the complex nature of regulation, including dodecameric (and tetradecameric) oligomerization with variable structures (Myers et al., 2017; Bayer and Schulman, 2019) and phosphorylation at ∼30 residues (Baucum et al., 2015).

Multisite phosphorylation is also the core mechanism for the regulation of ERK and SIK3. A role for multisite phosphorylation has been proposed to encode slower dynamics (Salazar et al., 2010). An increasing number of phosphorylation sites can create a time delay to reach a fully phosphorylated state. Even if phosphorylation at a specific residue is critical for the biological function, multisite phosphorylation can delay the timing of the specific phosphorylation by serving as a decoy (Kim and Ferrell, 2007). The multisite phosphorylation often occurs on the flexible region of a substrate protein, which may form intrinsically disordered regions (IDRs), and altered local electrostatic state produced by the multiple phosphorylation may greatly affect IDR structure (Iakoucheva et al., 2004; Wright and Dyson, 2015). These processes may collectively cause slow dynamics: excellent examples can be found in the regulation of the circadian 24-h timescale. A transcription factor, PER, in mammals/flies and its functional homolog FRQ in fungi undergo progressive multisite phosphorylation within a 24-h period. Many biochemical and genetic analyses have confirmed that progressive phosphorylation is important for the 24-h period of circadian oscillation (Gallego and Virshup, 2007).

PER and FRQ are typical proteins with IDRs, and biochemical studies indicate that the structures of PER and FRQ are significantly changed according to phosphorylation status. FRQ protein has a biased distribution of charged residues with an isoelectric point (pI) of the N terminal domain around 9.7 but that of the C terminal domain around 4.3. With such an uneven electrostatic distribution, FRQ protein is thought to be in a closed state in its natural form. Multisite phosphorylation on the N terminal domain equilibrates the pI of the two terminal domains and, through their repulsive interaction, opens the protein conformation (Querfurth et al., 2011). Like FRQ, it has been shown that PER in flies and mammals undergoes phosphorylation-dependent structural change (Chiu et al., 2011; Zhou et al., 2015). In addition, PER/FRQ and their responsible kinase CKI, as well as other binding partners, form a macromolecular complex (Aryal et al., 2017; Liu et al., 2019), the status of which (e.g., binding affinity of each component) may be changed slowly by multisite phosphorylation within the complex. In a sense, the slow dynamics produced by the collective and cooperative interaction of protein complexes driven by multisite phosphorylation may act as a timer to count the length of a 24-h period in eukaryotes (Larrondo et al., 2015; Dunlap and Loros, 2018; Ode and Ueda, 2018; Diernfellner and Brunner, 2020; Loros, 2020; Partch, 2020).

Turning back to the sleep control, the essential next step is to find the substrate protein and phosphorylation residues critical for the sleep-inducing effect of the sleep-promoting kinases. Because kinase is one of the best-studied protein class and there are sophisticated methodologies to find target substrates of each kinase and to perturb specific kinase activity such as engineered analog-sensitive kinase (Bishop et al., 2000; Lopez et al., 2014) and photo-switchable kinase inhibitor (Murakoshi et al., 2017). Do those kinases work in neurons in sleep control? If so, which brain area and subcellular region would it be? The substrates of sleep-promoting kinase should affect the neuronal firing pattern to induce the synchronized cortical slow oscillation: the direct substrates of sleep-promoting kinases might include ion channels and pumps that expressed cortex to modulate the neuronal membrane potential and also are highlighted their roles in the control of basal sleep duration and SWA by mice genetic studies (Espinosa et al., 2004; Lee et al., 2004; Anderson et al., 2005; Douglas et al., 2007; Sunagawa et al., 2016; Tatsuki et al., 2016; Yoshida et al., 2018; Muheim et al., 2019). Also, the kinases might indirectly affect the neuronal excitability by modulating the other enzymatic and cellular transport systems that control the level of neurotransmitters and neuromodulators. Upon the identification of the part of sleep-promoting kinase and substrate, further studies will illuminate the mechanism of how the simple transfer of the phosphoryl group governs the arousal level of mammals.

## Conclusion

The importance of kinases for the regulation of sleep has been supported by genetic and phosphoproteomic studies. Genetic studies indicated that CaMKII, SIK3, and ERK are potent kinases that induce sleep. Phosphoproteomic studies indicated that the sleep-wake cycle dominates the phosphorylation status of synaptic proteins. In particular, SIK3 appears to induce a similar phosphoproteomic profile to that caused by sleep deprivation, and the dynamics of CaMKII activation correlate well with the expected accumulation of sleep need. These results imply that kinases transduce and store cumulative neuronal activity as the phosphorylation status of their substates, including the phosphorylation status of the kinases themselves. In this view, the molecular entity of sleepiness is, at least in part, protein phosphorylation: we shall call this idea a phosphorylation hypothesis of molecular sleepiness–the kinase-centric understanding of sleep-wake transition may integrate the molecular mechanism of sleepiness and physiological functions of sleep to control protein activity.

So far, experimental data supporting this hypothesis have come primarily from studies of mouse models. Therefore, this hypothesis would be applicable to NREM sleep in mammals. However, since protein regulation by phosphorylation is conserved across species, and the sleep-promoting enzymes are conserved from vertebrates to invertebrates, it is worthwhile to investigate the extent to which the kinases and their substrates are involved in sleep regulation in other species as well. The possibility that the need for sleep can be estimated as a protein phosphorylation state suggests that the same index may be used to assess sleep status in species such as primitive invertebrates, where EEG cannot be used to measure sleep status. In combination with a rigorous evaluation of this hypothesis in mammalian sleep, it is important to investigate whether this hypothesis holds true in a variety of organisms. This should lead to a molecular exploration of the extent to which the regulatory mechanisms and functions of sleep are conserved across species.

## Author Contributions

KO and HU wrote the manuscript, conducted the literature survey, and approved the submitted version. Both authors contributed to the article and approved the submitted version.

## Conflict of Interest

The authors declare that the research was conducted in the absence of any commercial or financial relationships that could be construed as a potential conflict of interest.

## Footnotes

1 https://www.mousephenotype.org/data/genes/MGI:2387581
